# EK Sign: A Wrinkling of Uvula and the Base of Uvula in Obstructive Sleep Apnea-Hypopnea Syndrome

**DOI:** 10.1155/2015/749068

**Published:** 2015-11-29

**Authors:** Venkata Koka, Sandrine Baron, Darius Abedipour, Vincent Latournerie, Pierre El Chater

**Affiliations:** Sleep Study Laboratory, Department of Otolaryngology, Medical Center, 5 rue Docteur Pesqué, 93300 Aubervilliers, France

## Abstract

*Introduction*. Diagnosis of obstructive sleep apnea-hypopnea syndrome (OSAHS) is suspected in the presence of symptoms and/or pharyngeal alterations and skeletal abnormalities of maxilla and mandible. Our aim is to find a new clinical sign that leads to suspicion of OSAHS in snorers.* Methods*. We reviewed the clinical data of 69 snoring patients with or without OSAHS. We defined EK sign as the presence of horizontal wrinkling of uvula and the base of uvula and tried to correlate its presence with OSAHS.* Results*. EK sign was present in 25 of 69 patients. The positive predictive value of EK sign is 100%. The presence of EK sign significantly correlated with OSAHS (44% if AHI ≥ 5 and 0% if AHI < 5;* p* = 0.01) and severity of OSAHS (7% if AHI < 15 and 58% with AHI ≥ 15;* p* < 0.001).* Conclusions*. The EK sign is a strong predictor of OSAHS with a specificity of 100%. We recommend performing sleep tests in presence of EK sign in snorers even in the absence of other abnormalities or symptoms.

## 1. Introduction

Obstructive sleep apnea, a common disorder that affects about 2–4% of men and 1-2% of women of adult age (approximately 30 to 69 years), is characterized by repetitive episodes of complete or partial upper airway obstruction during sleep usually associated with snoring, intermittent hypoxemia, and sleep fragmentation.

The clinical picture may include one or more symptoms including snoring, nocturnal polyuria, excessive daytime sleepiness, morning headache, fatigue, neurocognitive deficits, personality alterations, and reduction of libido, irritability, depressive symptoms, and anxiety. Excessive daytime sleepiness is frequent and increases the risk of vehicles crashes and occupational accidents.

Obstructive sleep apnea-hypopnea syndrome (OSAHS) represents a complex alteration of the upper air passages, whose principal event corresponds to intermittent collapse of their walls in inspiration during sleep. The areas of obstruction can occur at isolated points or in multiple areas though their relation to sleep apnea has not been well established.

The suspicion of OSAHS should arise in presence of symptoms and physical examination in clinical practice before submitting the snoring patients to sleep studies such as polysomnography. The pharyngeal anatomy including lateral wall, tonsil, soft palate, uvula, and tongue volume and skeletal characteristics of maxilla and mandible have been studied to identify the predictive signs of OSAHS [1, 2].

In our study we tried to identify a morphological alteration of the soft palate in snorers as predictor of presence and severity of OSAHS.

## 2. Material and Methods

This a retrospective of 69 consecutive patients for snoring with or without OSAHS between 2012 and 2014 seen at the Sleep Study Laboratory at the Department of Otolaryngology, Medical Center, Aubervilliers, France.

Inclusion criteria were the presence of snoring with or without symptoms related to OSAHS. All patients underwent a clinical evaluation for a clinical history of hypertension, other cardiovascular diseases, diabetes mellitus, hypothyroidism, and completed Epworth Sleepiness Score. The patient's sex, age, and BMI were noted.

**Figure 1:**
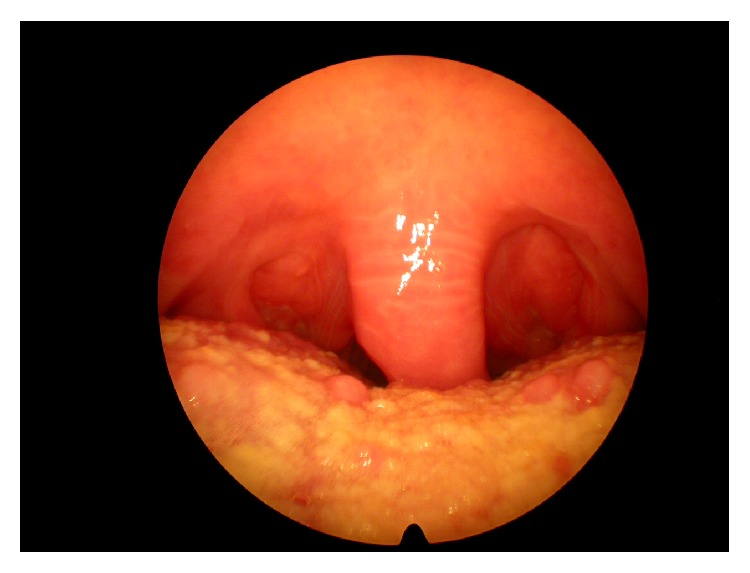
Wrinkling of uvula (EK sign) in obstructive sleep apnea-hypopnea syndrome.

**Figure 2:**
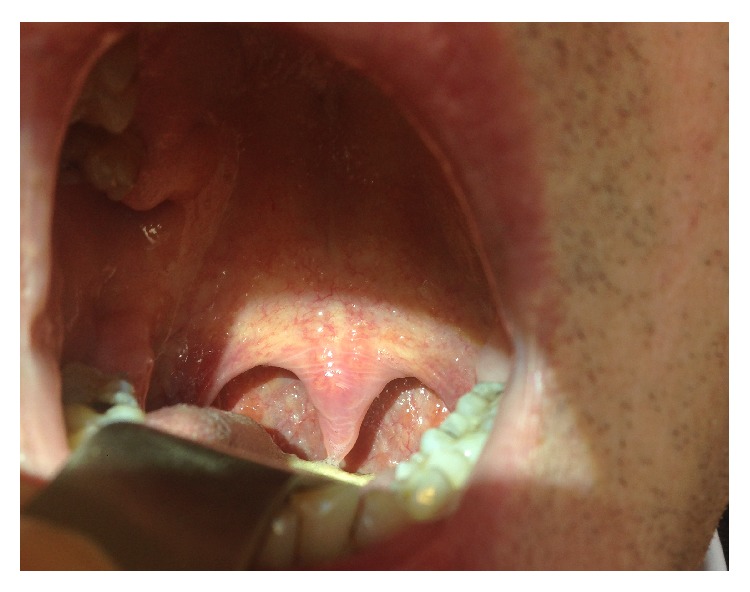
Wrinkling of uvula and the base of uvula (EK sign) in obstructive sleep apnea-hypopnea syndrome.

The oropharyngeal evaluation is carried out to look for the presence of horizontal wrinkling of the uvula and the base of uvula (Figures 1 and 2). We named this morphological alteration as EK sign (El Chater and Koka sign).

All the patients subsequently underwent a polygraphy (PG) examination to diagnose OSAHS. The PG parameters were nasal airflow limitations, thoracic and abdominal movements, and oxygen saturation by pulse oximetry.

Obstructive apnea was defined as a ≥10-second cessation of airflow on the pressure nasal cannula; hypopnea was defined as a ≥50% reduction in airflow or a <50% airflow reduction on the nasal pressure cannula accompanied by a ≥3% decrease in arterial oxyhemoglobin saturation (SpO_2_) recorded using finger pulse oximetry.

Depending on apnea and hypopnea index (AHI), the patients are distributed into four groups in this study: snorers without OSAHS when AHI is less than 5, mild OSAHS when AHI was equal to 5 to 15, moderate OSAHS when AHI was equal to 15 to 30, and severe OSAHS when AHI is greater than 30.

The association of EK sign with AHI and BMI is determined by chi-square test with Yates correction. The* p* value less than or equal to 0.05 is considered significant. The sensitivity and specificity of EK sign are calculated.

## 3. Results

Sixty-nine patients were included in this study; 27 were female and 42 were male. The age ranged from 22 to 74 with a mean patient age of 49. The EK sign was found in 25 of 69 (36%) patients in our series.

The patients were distributed according to BMI in 5 groups (group I: 18.5–24; group II: ≥25–29; group III: ≥30–34; group IV: ≥35–40; group V: >40). The BMI ranged from 21 to 48 kg/m^2^ (mean 31, median 30) and no significant correlation was found between presence of EK sign and degree of BMI. The EK sign was present in 5 of 12 patients (41.6%) in group I, 4 of 21 (19%) patients in group II, 10 of 21 patients (47.6%) in group III, 4 of 6 patients in group IV (40%), and 2 of 9 patients (22%) in group V (*p* > 0.05).

All the patients were snorers and had ambulatory polygraphy with a mean AHI score ranging from 0 to 80 per hour (mean 25, median 22). According to degree of AHI, 12 patients (17.3%) were simple snorers without OSAHS, 17 patients (24.6%) had mild OSAHS, 15 patients (21.7%) had moderate OSAHS, and 25 patients (32.2%) had severe OSAHS (Table 1).

The presence of EK sign significantly correlated with the presence of OSAHS; EK sign was positive in 25 of 57 (44%) patients with sleep apnea and absent (0%) in all the 12 patients with no apnea (*p* = 0.01). The presence of EK sign significantly correlated with the severity of OSAHS: 58% (23 of 40) of patients with AHI ≥ 15 compared to 7% (2 of 29) if AHI is less than 15 (*p* < 0.001) (Table 2).

Of 69 patients, we noted 25 true positives (TP), 0 false positives (FP), 12 true negatives (TN), and 32 false negatives (FN). The specificity (true negative rate) and sensitivity (true positive rate) of EK sign are 100% and 44%, respectively. The positive predictive value (PPV) of EK sign was 100%; 25 of 69 patients showed EK sign and all of them presented OSAHS whereas EK sign is absent in all 12 patients with no apnea. The negative predictive value (NPV) of EK sign was 27%; EK sign was absent in 32 of 57 patients (56%) with OSAHS.

## 4. Discussion

Diagnosis of OSAHS is often suspected in the presence of one or more symptoms such as snoring, nocturnal gasping, daytime fatigue, and somnolence before proceeding to appropriate sleep studies. The predictive criteria based on subjective symptoms such as Epworth scoring are not conclusive in the literature. There is a need to look for the objective physical signs to suspect the presence of OSAHS.

Friedman et al. [3] identified tonsil size, modified Mallampati classification (MMC), and body mass index as predictors of OSAHS. Zonato et al. [2] studied pharyngeal characters including the tonsil size, abnormal soft palate, abnormal uvula, voluminous lateral wall, and web palate and found a statistically significant correlation between the presence of ogival palate and sleep apnea. Woodson and Naganuma [4] noted that AHI correlated with BMI and posterior wall redundancy. It remains a challenge for clinicians to find a pathognomic sign or multiple signs that leads to a high suspicion of OSAHS.

Morphological alterations in the soft palate observed in cephalometric studies of normal and apneic individuals have been reported in the literature. You et al. described six anatomical variations according to velar dimensions in nonapneic population: type 1, leaf shaped; type 2, rat tailed shaped; type 3, butt like; type 4, straight line shaped; type 5, S or distorted shaped; type 6, crooked appearance [5]. Pépin et al. [6] found that the presence of a hooked appearance or type 5 S shaped soft palate indicated a high risk of OSAHS. The hooking of soft palate was a result of angulations of 30° between the distal part of uvula and longitudinal axis soft palate and this might cause sudden and major reductions in the oropharyngeal dimensions, thus increasing the upper airway resistance and transpharyngeal gradient thereby causing pharyngeal collapse. However, the snoring patients are not systematically submitted to cephalometric studies and there is a necessity to identify clinical signs such as EK sign that lead to suspicion of OSAHS.

The OSAHS results in histological changes in the pharyngeal fibromuscular tissues that might cause morphological alterations of pharyngeal soft tissues. Smirne et al. [7] demonstrated in their histological studies on middle constrictor that apneic individuals have an abnormal distribution of muscle fibers, with reduction of type I and type IIb fibers and an increase in type IIa fibers. In a histological study of the soft palate of apneic individuals, Woodson et al. [8] found hypertrophy of mucosal glands, edema of the lamina propria, and atrophy of musculature and demyelinization of peripheral nerve fibers. Bastos et al. [9] found increased quantity of elastic fibers and collagen fibers in the extracellular matrix in the soft palate in OSAHS patients compared to nonapneic patients and also observed structural changes in palatoglossal muscle.

We believe that the morphological alteration such as EK sign with horizontal wrinkling of uvula may be the result of histological changes produced by OSAHS. It is difficult to establish the role of trauma induced by vibrations of soft palate in snoring individuals, as EK sign is absent in all simple snorers without apnea.

We observed a significant correlation of EK sign with the presence of OSAHS (*p* = 0.01) and the grade of AHI (*p* < 0.001). We found EK sign as the pathognomic clinical sign of OSAHS, with a strong positive predictive value of 100% and a specificity of 100%. EK sign was typically absent in snorers with no apnea. However, we should be cautious in interpretation of absent EK sign, as we found a false negative rate of 56% in apneic individuals in our study.

## 5. Conclusions

The wrinkling of uvula and the base of uvula (EK sign) alone is a strong predictor of OSAHS in snoring patients seen at clinical practice during a standard otolaryngological examination. Hence we recommend performing sleep tests in presence of EK sign directly even in the absence other physical and skeletal abnormalities or without any symptoms of sleep apnea.

## Figures and Tables

**Table 1:** EK sign according to AHI.

Apnea and hypopnea index (AHI)	EK sign present	EK sign absent
AHI < 5 (snorers without OSAHS)	0 (0%)	12 (100%)
AHI ≥5 to <15 (mild OSAHS)	2 (12%)	15 (88%)
AHI ≥15 to <30 (moderate OSAHS)	7 (47%)	8 (53%)
AHI ≥ 30 (severe OSAHS)	16 (64%)	9 (36%)

OSAHS: obstructive sleep apnea-hypopnea syndrome.

**Table 2:** EK sign according to presence and severity of OSAHS.

Degree of OSAHS	EK sign present	EK sign absent	*p* value
No OSAHS	0 (0%)	12 (100%)	
OSAHS	25 (44%)	32 (56%)	*p* = 0.01
None to mild OSAHS (AHI < 15)	2 (7%)	27 (93%)	
Moderate or severe OSAHS (AHI > 15)	23 (58%)	17 (42%)	*p* < 0.001

OSAHS: obstructive sleep-hypopnea syndrome; AHI: apnea and hypopnea index.
